# The Na^+^,K^+^-ATPase in complex with beryllium fluoride mimics an ATPase phosphorylated state

**DOI:** 10.1016/j.jbc.2022.102317

**Published:** 2022-08-02

**Authors:** Marlene U. Fruergaard, Ingrid Dach, Jacob L. Andersen, Mette Ozol, Azadeh Shahsavar, Esben M. Quistgaard, Hanne Poulsen, Natalya U. Fedosova, Poul Nissen

**Affiliations:** 1 Department of Molecular Biology and Genetics, DANDRITE - Nordic EMBL Partnership for Molecular Medicine, Aarhus University, Aarhus C, Denmark; 2 Department of Biomedicine, Aarhus University, Aarhus C, Denmark

**Keywords:** Na^+^,K^+^-ATPase, beryllium fluoride, E1–E2, inward-outward, transporter, P-type ATPase, membrane protein, crystallography, BeF, beryllium fluoride, MR, molecular replacement, NCS, noncrystallographic symmetry, OBN, ouabain, PDB, Protein Data Bank, SERCA, sarco/endoplasmic reticulum Ca^2+^-ATPase, TM, transmembrane

## Abstract

The Na^+^,K^+^-ATPase generates electrochemical gradients of Na^+^ and K^+^ across the plasma membrane *via* a functional cycle that includes various phosphoenzyme intermediates. However, the structure and function of these intermediates and how metal fluorides mimick them require further investigation. Here, we describe a 4.0 Å resolution crystal structure and functional properties of the pig kidney Na^+^,K^+^-ATPase stabilized by the inhibitor beryllium fluoride (denoted E2–BeF_x_). E2–BeF_x_ is expected to mimic properties of the E2P phosphoenzyme, yet with unknown characteristics of ion and ligand binding. The structure resembles the E2P form obtained by phosphorylation from inorganic phosphate (P_i_) and stabilized by cardiotonic steroids, including a low-affinity Mg^2+^ site near ion binding site II. Our anomalous Fourier analysis of the crystals soaked in Rb^+^ (a K^+^ congener) followed by a low-resolution rigid-body refinement (6.9–7.5 Å) revealed preocclusion transitions leading to activation of the dephosphorylation reaction. We show that the Mg^2+^ location indicates a site of initial K^+^ recognition and acceptance upon binding to the outward-open E2P state after Na^+^ release. Furthermore, using binding and activity studies, we find that the BeF_x_-inhibited enzyme is also able to bind ADP/ATP and Na^+^. These results relate the E2–BeF_x_ complex to a transient K^+^- and ADP-sensitive E∗P intermediate of the functional cycle of the Na^+^,K^+^-ATPase, prior to E2P.

Na^+^,K^+^-ATPase maintains physiological concentrations and gradients of Na^+^ and K^+^, which are crucial for animal cells. The enzyme is a binary complex of a large α-subunit of the P-type ATPase family responsible for ion transport and enzymatic reaction and a β-subunit acting as a chaperone and functional modulator. Furthermore, regulatory FXYD proteins may associate with the complex in a tissue-dependent manner. The ion exchange is driven by ATP hydrolysis *via* enzyme autophosphorylation and dephosphorylation, and a minimal scheme of the Na^+^,K^+^-ATPase cycle, also termed the Post-Albers scheme, includes two major conformations, E1 and E2, in their phosphorylated and nonphosphorylated states (1, 2). These four states display different ion-binding affinities and provide access to the ion sites either from inward/cytoplasmic or outward/extracellular sides of the membrane. The dephosphorylation of the phosphoenzyme pool (preformed by Na^+^-dependent phosphorylation) in response to addition of either ADP or K^+^ was shown to be biphasic in both cases, in agreement with the presence of two phosphointermediates in the reaction cycle (E1P and E2P). The amplitude of the fast phase of P_i_ release in ADP- or K^+^-chase experiments reflected respective fractions of ADP-sensitive (E1P) and K^+^-sensitive (E2P) phosphoenzymes in the Post-Albers scheme. However, the sum of these two phosphointermediates considerably exceeded the amount of initially phosphorylated enzyme (3), exposing insufficiency of a two-state model (E1P/E2P) for phosphointermediates. The number and nature of the phosphoenzyme conformations have therefore been debated, and a consensus was found in the existence of three phosphoenzymes: ADP-sensitive K^+^-insensitive (E1P), ADP- and K^+^-sensitive (E∗P), and ADP-insensitive K^+^-sensitive (E2P) phosphoenzymes (3, 4, 5). Similar dephosphorylation phenomena were also observed for the closely related sarco/endoplasmic reticulum Ca^2+^-ATPase (SERCA) (4).

Recent success in crystallization revealed structures mimicking several native phosphointermediates of the Na^+^,K^+^-ATPase. Protein complexes with metal fluorides (*i.e.*, beryllium, aluminium, and magnesium fluorides, denoted BeF_x_, AlF_x,_ and MgF_x_ because of varying degrees of water coordination and therefore different fluoride stoichiometries and net charges) mimic different states of phosphoryl transfer reactions and phosphointermediates. Thus, a MgF_x_ complex resembles an [K_2_]E2∙P_i_ state with occluded K^+^ and noncovalently bound phosphate (P_i_), whereas E1∙AlF_4_^−^∙ADP contains three occluded Na^+^ ions and represents an intermediate leading to the [Na_3_]E1P-ADP phosphoenzyme (6, 7). The BeF_x_ complex of the Na^+^,K^+^-ATPase (8, 9) is structurally similar to the P_i_-induced (“backdoor” phosphorylated) E2P phosphoenzyme stabilized by cardiotonic steroids (10, 11) and E2P^ATP^ formed by ATP in the forward reaction (12).

The flexibility and intermediary functional properties of the BeF_x_ complex were anticipated earlier from the presence of a characteristic H^+^ leak current mediated by Na^+^,K^+^-ATPase (13) and in SERCA from the measurements of Ca^2+^ occlusion (14) and Ca^2+^-mediated reactivation (15).

Here, we describe functional properties of the E2–BeF_x_ complex of Na^+^,K^+^-ATPase and structural rearrangements in its crystal structure induced by binding of Rb^+^ as a K^+^ congener. Close structural resemblance to the P_i_-induced E2P phosphoenzyme stabilized by cardiotonic steroids (10, 11) includes outward-open ion-binding sites, which prompted investigations of the effect of extracellular Mg^2+^.

Notably, we also find that BeF_x_ dissociation from the enzyme is accelerated by both nucleotide and ion binding—these are characteristics that relate the E2–BeF_x_ structure to the ADP- and K^+^-sensitive (E∗P) phosphoenzyme.

## Results

### Formation of the BeF_x_ complex

The BeF_x_-inhibited complex of Na^+^,K^+^-ATPase was formed by preincubation of the membraneous enzyme in 20 mM histidine (pH 7.0), 10 mM NaF, 0.5 mM MgCl_2_, 20 μM BeSO_4_, and used in all biochemical studies as well as initial material for crystallization.

### Overall crystal structure of the BeF_x_ complex of the Na^+^,K^+^-ATPase

**Figure 1:**
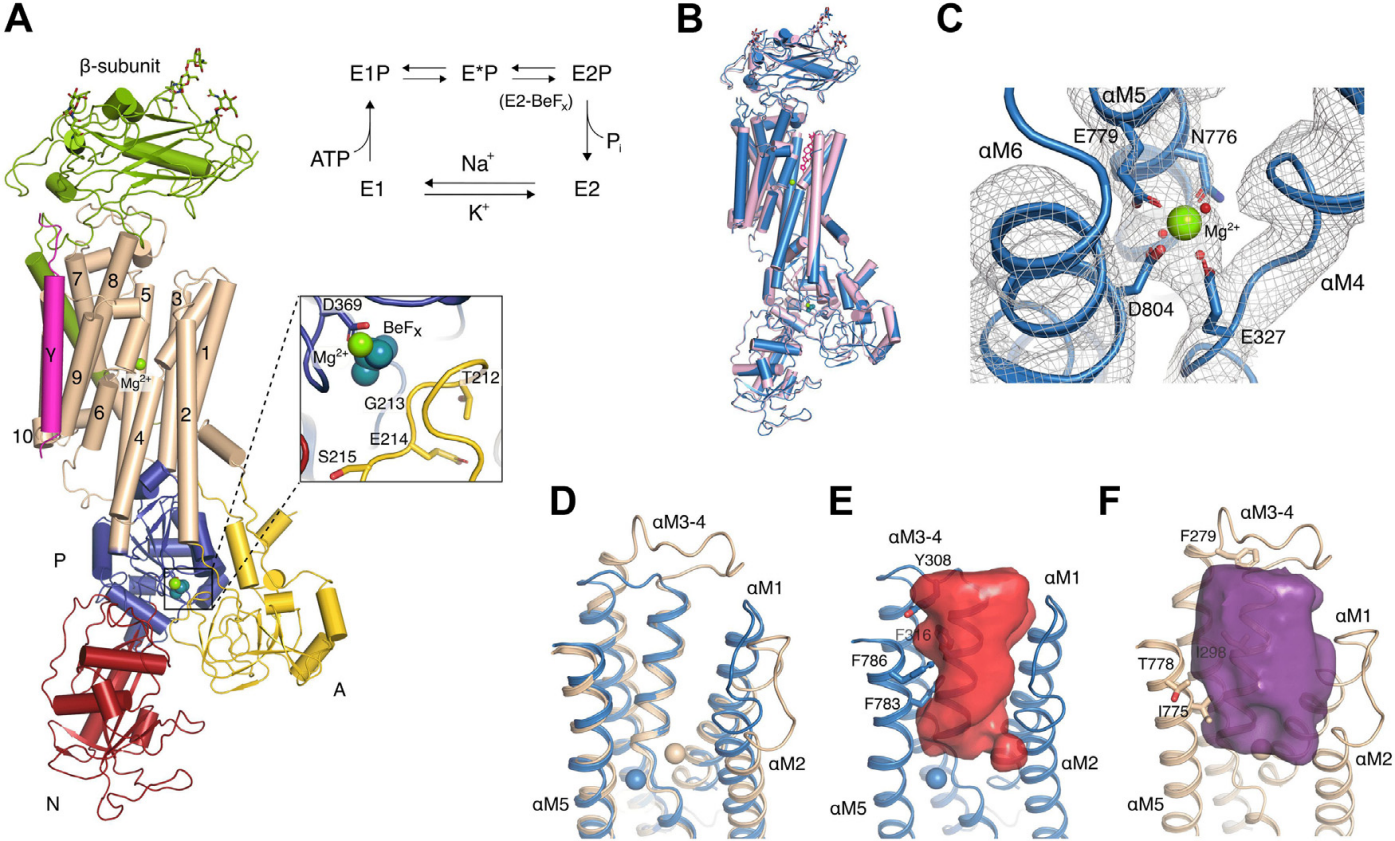
**Crystal structure of E2–BeF**_**x**_**state of Na**^**+**^**,K**^**+**^**-ATPase.***A*, *cartoon* representation of E2–BeF_x_ state colored according to the different domains: *red* (nucleotide-binding [N] domain), *blue* (phosphorylation [P] domain), *yellow* (activator [A] domain), *wheat* (transmembrane [TM] domain, αM1–M10), *green* (β-subunit), and *hot pink* (γ-subunit). Close-up view of the phosphorylation site is shown in the *inset*. Mg^2+^ ions and the BeF_x_ bound to Asp369 are depicted as *green* and *teal spheres*, respectively. The Post-Albers reaction scheme of Na^+^,K^+^-ATPase accumulating 3-pool model of phosphoenzymes is shown in the *upper right*. *B*, overall comparison of E2–BeF_x_ (*blue*) and ouabain-bound P_i_-induced E2P (*pink*). Mg^2+^ ions are depicted as *green spheres*, and ouabain is colored in *hot pink*. The structures were aligned on the TM segment αM7–10. *C*, coordination of Mg^2+^ by Glu327 (M4), Asn776, Glu779 (M5), and Asp804 (M6) side chains. Mg^2+^ and water are depicted as *green* and *red spheres*, respectively. Density map contoured at 1.5 σ. *D*, extracellular access channel of Na^+^,K^+^-ATPase E2–BeF_x_ (*blue*) aligned with SERCA E2–BeF_x_ (PDB ID: 3B9B (15), *wheat*). The structures were aligned by αM5–M6. Bound Mg^2+^ ions are shown as *spheres* also in *blue* and *wheat*. *E* and *F*, water cavity representation of Na^+^,K^+^-ATPase E2–BeF_x_ (*red*) and SERCA E2–BeF_x_ (*purple*), showing a narrower entrance to the ion-binding sites in the Na^+^,K^+^-ATPase E2–BeF_x_ complex. Cavities were calculated in HOLLOW (49). PDB, Protein Data bank; SERCA, sarco/endoplasmic reticulum Ca^2+^-ATPase.

The crystal structure of pig kidney Na^+^,K^+^-ATPase (α_1_, β_1_, and FXYD2 also known as γ) was determined by molecular replacement (MR) using the ouabain-bound P_i_-induced (E2P-OBN) form as the starting model (Protein Data Bank [PDB] ID: 4HYT) (10), and at later stage compared with the structure of a different crystal form of the E2–BeF_x_ complex (PDB ID: 7D91 (9)). The final model was refined against anisotropically truncated data extending to 4.05 Å, resulting in *R*_work_ and *R*_free_ values of 22.7% and 27.6%, respectively (Fig. 1*A*, Table 1).

**Table 1:** Data collection and model refinement statistics

Parameters	Native crystal		Rb^+^ soak	
	Elliptical truncation	Spherical truncation	10 mM (20 s)	50 mM (3 h)
Wavelength (Å)	1.0	1.0	0.814	0.814
Resolution range (Å)	29.7–4.1 (4.2–4.05)	29.7–4.1 (4.2–4.05)	33.5–7.5 (7.7–7.5)	30.9–6.9 (7.2–6.9)
Space group	P2_1_2_1_2_1_	P2_1_2_1_2_1_	P2_1_2_1_2_1_	P2_1_2_1_2_1_
Unit cell parameters a, b, and c (Å)	117.5, 118.1, 494.7	117.5, 118.1, 494.7	119, 119.2, 498.2	118.9, 118.9, 496.8
Total reflections	1,494,985	1,503,991	136,605	172,233
Unique reflections	56,729	57,347	9354	11,882
Multiplicity	26.1	26.2	14.0	14.3
*I*/σ(*I*)^a^	10.7 (2.0)	7.3 (0.7)	6.5 (1.3)	7.6 (1.3)
Completeness (%)	68.5 (5.5)	99.5 (99.6)	99.6 (99.2)	99.6 (97.8)
Mn(I) half-set correlation CC1/2 (%)	98.8 (60.5)	99.8 (26.3)	99.9 (60.3)	99.8 (0.79)
*R*_merge_ (%)^a^	28.9 (180.4)	44.6 (597)	31.2 (214)	30.31 (205.3)
*R*_pim_ (%)^b^	5.9 (37.9)	8.9 (122)	10.6 (60.2)	8.3 (56.3)
Wilson *B*	148^c^		429	290
Refinement statistics				
Resolution range (Å)	29.7–4.1		25–7.5	25–6.9
Reflections	39,148		9346	11,849
*R*/*R*_free_ (%)	22.7/27.6		29.4/33.8	28.5/31.8
rmsds				
Bonds (Å)	0.014		0.014	0.016
Angles (°)	1.35		1.54	1.56
Average *B* values				
Protein (Å2)	190		480	410
Ligands (Å2)	198		553	440
Ramachandran plot				
Favored (%)	93.8		81.9	84.8
Allowed (%)	6.1		14.8	12.2
Outliers (%)	0.1		3.3	3.0

Values in parentheses are for the highest resolution shell.

a $R_{\text{merge}}={\text{Σ}}_{h}{\text{Σ}}_{i}\left|I_{hi}-\left\langle I_{h}\right\rangle \right|/{\text{Σ}}_{h}{\text{Σ}}_{i}\left\langle I_{h}\right\rangle$.

b $R_{\text{pim}}={\text{Σ}}_{h}{\text{Σ}}_{i}{\left(\frac{1}{n_{h}-1}\right)}^{1/2}\left|I_{hi}-\left\langle I_{h}\right\rangle \right|/{\text{Σ}}_{h}{\text{Σ}}_{i}\left\langle I_{h}\right\rangle$, where *n*_*h*_ is the multiplicity, *I*_*hi*_ is the *i*th intensity of reflection *h*, and $\left\langle I_{h}\right\rangle$ is the weighted average intensity for all observations *i* of reflection *h*.

c The Wilson plot had a linear appearance in the 4 to 5 Å resolution range, from which a Wilson *B* was derived. For the low-resolution datasets, the Wilson *B* is not very meaningful.

BeF_x_ coordinated to the conserved Asp369 phosphorylation site in the conserved DKTG segment of the P domain was identified in unbiased initial *F*_o_–*F*_c_ difference map. Comparison shows similar arrangements of the P domains in both the P_i_-induced OBN-bound E2P state (10) and in the present BeF_x_ structure. The TGES loop of the actuator (A) domain is in close proximity to BeF_x_ and protects the phosphorylation site from spontaneous hydrolysis (Fig. 1*A*). High structural similarity with E2P-OBN (rmsd = 0.59 Å for all C_α_), also reveal subtle OBN-induced rearrangements in the αM1–M4 segments of the earlier reported E2P-OBN structure. Slight outward tilting of the αM1–M2 bundle is due to the extensive hydrogen-bonding network between the β-surface of OBN and polar side chains of αM1, αM2, and αM6 (10). Furthermore, the inhibitor-bound site makes the extracellular part of the αM3–M4 helices close in, causing a ∼4° tilt of αM4 with pivot at Val322 (Fig. 1*B*) as compared with E2–BeF_x_.

The extracellular ion pathway is formed by the transmembrane (TM) αM1–M6 helices, which define an outward-open arrangement similar to SERCA1a in the Mg^2+^-stabilized E2–BeF_x_ form (15) (Fig. 1*D*). The arrangement of αM2–M5 helices in Na^+^,K^+^-ATPase, however, is slightly more compact, and the residues lining the pathway have larger hydrophobic side chains (*e.g.*, Tyr308, Phe316, Phe783, and Phe786 in Na^+^,K^+^-ATPase corresponding to Phe279, Ile298, Ile775, and Thr778 in SERCA) (Fig. 1, *E* and *F*). The extracellular ion pathway is thus narrow but solvated, consistent with a low voltage sensitivity of K^+^ binding through such a pathway (16) and a voltage-insensitive release of the third and last Na^+^ ion from the Na^+^-bound state (17).

For the cation-binding sites in the TM domain, we observed a residual density near site II in the initial unbiased *F*_o_–*F*_c_ difference map, similar to a Mg^2+^ site in the E2P–OBN complex (10). Indeed, the E2–BeF_x_ crystals were grown in the presence of 175 mM MgCl_2_, and Mg^2+^ binding is likely to stabilize an open conformation as seen also for the E2–BeF_x_ complex of SERCA1a (15), although the site is shifted (Fig. 1*D*). The Mg^2+^ site seems to overlap with incoming K^+^ (Fig. 1*C*) (see further).

### P_i_-induced E2P and E2–BeF_x_ complexes have similar functional properties

**Figure 2:**
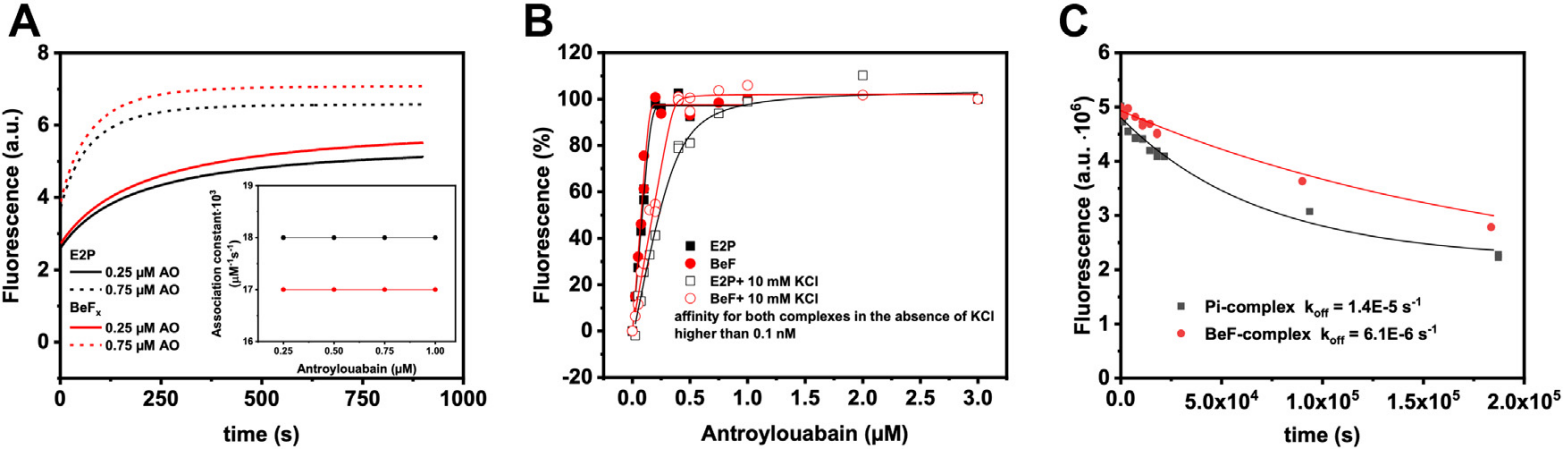
**Interaction of P**_**i**_**-induced E2P and BeF**_**x**_**complexes of Na**^**+**^**,K**^**+**^**-ATPase with anthroylouabain.** Data obtained with P_i_-induced E2P are shown in *black color*, with BeF_x_ complex in *red*. *A*, changes in anthroylouabain (AO) fluorescence at two different concentrations, 0.25 μM (*full line*) and 0.75 μM (*stippled line*), were induced by its binding to the E2P and BeF_x_ complexes. Calculated second-order association rate constants for the reactions are shown in the *inset*. *B*, amplitude of the fluorescence change as function of AO concentration in the absence or the presence of 10 mM KCl. *C*, time course of AO dissociation from E2P and BeF_x_ complexes.

Structural similarities between P_i_-induced E2P and E2–BeF_x_ complexes (Fig. 1*B*) are also supported by their functional properties, for example, by interactions with cardiotonic steroids. Figure 2 summarizes kinetics of anthroylouabain binding to these complexes, which, judged from the crystallographic data, have very similar organization of the CTS-binding cavities. The main conclusion from the kinetic experiments is that affinities of both forms to anthroylouabain are very high, with only small numeric differences in the values of association and dissociation rate constants. Although the effect of K^+^ on anthroylouabain affinity seems to be less pronounced for E2–BeF_x_, it is still in the direction of decreased affinity for both forms.

### Interactions of Na^+^, K^+^/Rb^+^, and Mg^2+^ with the E2–BeF_x_ complex

The E2–BeF_x_ crystal structure reveals that the cation-binding cavity is open to the extracellular side and occupied by a Mg^2+^ ion. The homologous complex of SERCA in crystallized form also contained Mg^2+^ (15) (Fig. 1*F*), whereas in functional studies, it was shown to bind and occlude Ca^2+^ (14). Do the ions bind to the E2–BeF_x_ complex of Na^+^,K^+^-ATPase, and what are the functional consequences of this binding? We investigated the interactions with Na^+^, K^+^ (Rb^+^ as congener), and Mg^2+^.

### Mg^2+^ binding does not affect Na^+^ and K^+^ binding

The effect of extracelluar Mg^2+^ on binding of Na^+^, K^+^, and H^+^ was investigated by two-electrode voltage clamping on *Xenopus laevis* oocytes (a well-established model to study functional properties of the Na^+^,K^+^-ATPase), expressing human α_2_β_1_ (see sequence alignment, Fig. S1). In the absence of extracellular K^+^, the pump is distributed between the whole range of phosphoenzymes because of voltage-dependent binding and release of Na^+^. We assayed Na^+^ binding in the presence of 0, 1, and 5 mM Mg^2+^ and found no difference in either apparent affinity for Na^+^ (Q/Vm curves) or the rate constants of release (Fig. S2, *A* and *B*). We next examined the potency and efficacy of K^+^ binding by measuring the steady-state current at −30 mV in the presence of 115 mM Na^+^ with or without 5 mM Mg^2+^ at varying K^+^ concentrations. Again, there were no differences in currents suggesting that physiological concentrations of extracellular Mg^2+^ have no significant effects on binding of Na^+^ or K^+^ from the extracellular environment (Fig. S2, *C* and *D*).

**Figure 3:**
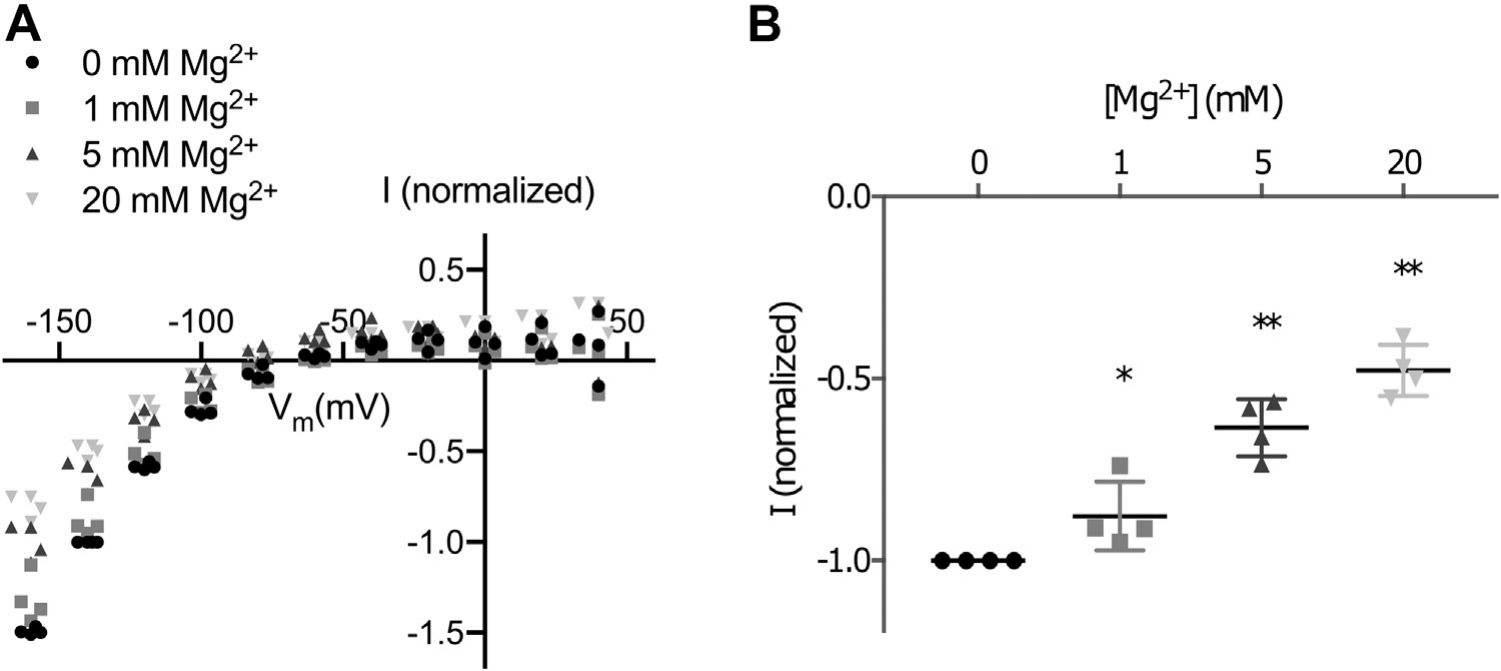
**Inward leak current is affected by magnesium.***A*, the ouabain-sensitive steady-state current measured in the absence of extracellular sodium and potassium in response to membrane potential for different concentrations of magnesium. Current is normalized to −1 for 0 mM Mg^2+^ at −140 mV (n = 4). *B*, leak currents at −140 mV. For 5 and 20 mM, the currents are significantly smaller. *Line* at mean with SD (∗*p* < 0.05; ∗∗*p* < 0.0001; Student’s *t* test).

However, in the absence of extracellular Na^+^ and K^+^, an inwardly rectifying H^+^ leak current is observed for the Na^+^,K^+^-ATPase (7), in particular at hyperpolarized potentials. This inward leak, however, was significantly inhibited at hyperpolarized potentials by 5 mM or 20 mM Mg^2+^ (Fig. 3).

Thus, the affinity for the extracellular Mg^2+^ is so low that it does not interfere with Na^+^ or K^+^ binding under physiological conditions. Yet, when bound, it stabilizes the outward-open state and interferes with a H^+^ leak current.

### Beryllium fluoride prevents oligomycin-induced Na^+^ occlusion

**Figure 4:**
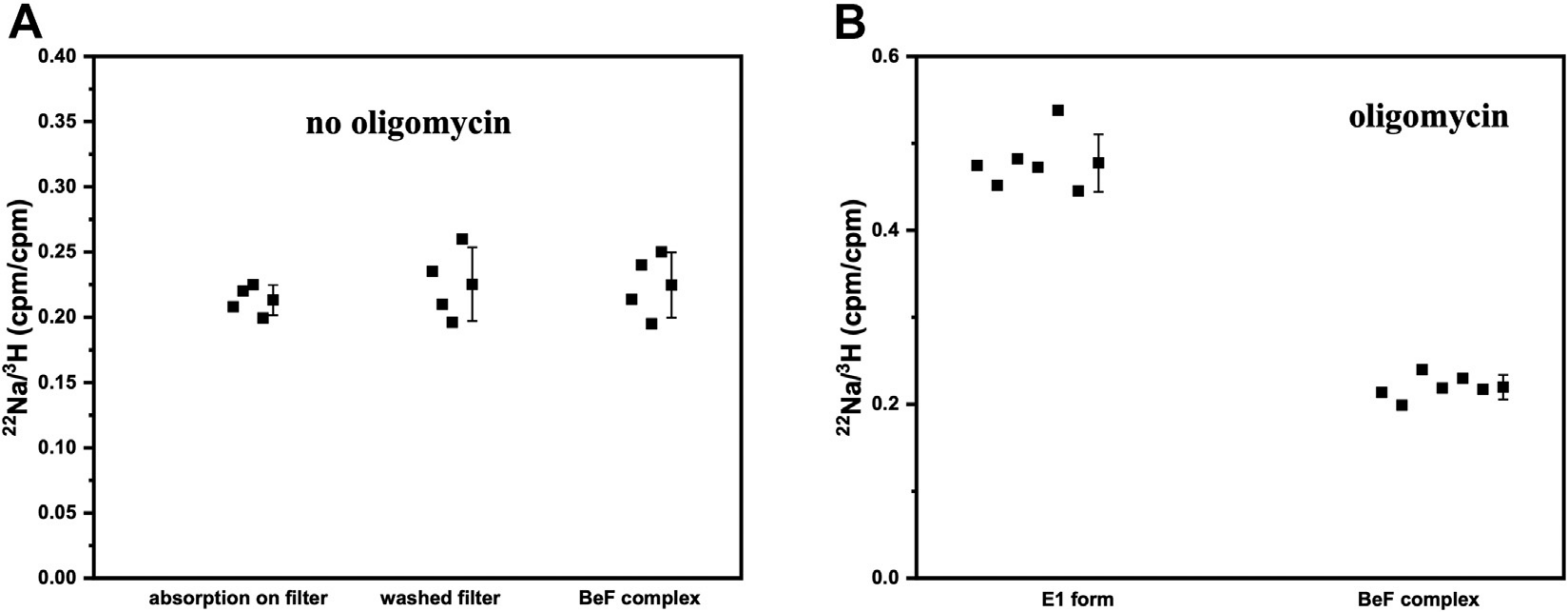
**BeF**_**x**_**binding prevents Na**^**+**^**occlusion in the presence of oligomycin.** Counts for ^22^Na^+^ on the filter are related to the counts for ^3^H^+^ used as internal standard for the nonspecific binding. The ^22^Na^+^/^3^H^+^ ratio (counts per filter) after filtration of 0.4 ml incubation media are shown for the samples of different compositions. *A*, in the absence of added oligomycin: (1) no protein, nonspecific binding without washing of the filter; (2) no protein, nonspecific binding after washing of the filter; (3) Na^+^,K^+^-ATPase in the E2–BeF_x_ form, followed by washing of the filter. The ratio was not changed by washing, indicating that both isotopes interact with filter in the same way. It was not affected by the presence of BeF_x_ complex of the Na^+^,K^+^-ATPase. *B*, in the presence of oligomycin. (1) Na^+^,K^+^-ATPase in E1 conformation, followed by washing of the filter; (2) Na^+^,K^+^-ATPase in the E2–BeF_x_ form, followed by washing of the filter. The data from individual experiments as well as mean values ± SD are shown.

The E2–BeF_x_ form of SERCA1a is capable of Ca^2+^ occlusion (14). We therefore investigated Na^+^ occlusion by the E2–BeF_x_ form of the Na^+^,K^+^-ATPase but failed to reveal any bound Na^+^ (Fig. 4*A*). We repeated the experiment in the presence of oligomycin, which decreases the rate of Na^+^ release (18, 19) and found an expected increase in ^22^Na^+^ bound to the E1 state, where the Na^+^,K^+^-ATPase occluded approximately 2.5 nmol per nmol protein (Fig. 4*B*). However, under the conditions of BeF_x_ complex formation, the ^22^Na^+^/^3^H^+^ ratio was the same as in the absence of enzyme or oligomycin (Fig. 4*A*), implying that binding of BeF_x_ effectively prevented Na^+^ occlusion by the enzyme.

### K^+^ occlusion by the BeF_x_ complex

**Figure 5:**
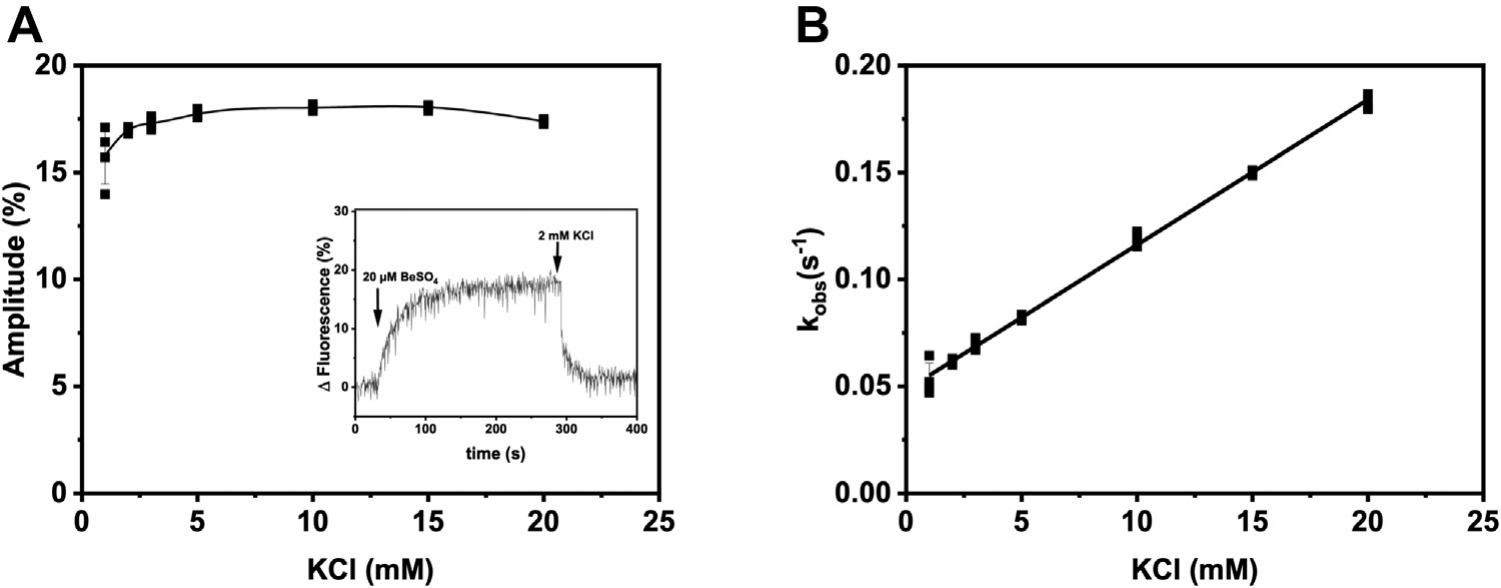
**Interactions of K**^**+**^**and BeF**_**x**_**complex monitored by RH421 fluorescence.***Inset* (*A*) illustrates changes in RH421 fluorescence in response to addition of ligands to pig kidney enzyme expressed as percentage of the initial level of fluorescence. The K-induced responses were fit with a monoexponential function. Data from 3 to 4 individual experiments as well as the mean value ± SD are shown in *A* and *B*. *A*, the amplitude of the fluorescence change induced by addition of varying K^+^-concentration to preformed Na^+^,K^+^-ATPase–BeF_x_ complex. *B*, the observed rate constant of the fluorescence change (*k*_obs_) as function on K^+^ concentration.

BeF_x_ binding to Na^+^,K^+^-ATPase is associated with an increase of RH421 fluorescence, similar to the response of this dye to enzyme phosphorylation, whereas addition of K^+^ ions induces a decrease in fluorescence (Fig. 5*A*, *inset*, and (20)). The values for both amplitude and *k*_obs_ for K-response extracted by fitting with a monoexponential function show a hyperbolic concentration dependence for the amplitude of fluorescence that decrease from its maximal level already at 2 mM KCl and a linear increase for *k*_obs_ (Fig. 5). Knowing that K^+^ interacts with P_i_-induced E2P in the same way (21), we assumed that direct binding of K^+^ is followed by its occlusion in E2–BeF_x_ form. Indeed, direct measurements with ^86^Rb^+^ (as K^+^ congener) showed its accumulation in the E2–BeF_x_ form (20).

### Rb^+^ binding and extracellular gate closure

Comparison of the E2–BeF_x_ (this study) and [Rb_2_]E2–MgF_x_ (6) states reveals the overall transitions of the extracellular gate closure upon K^+^/Rb^+^ binding. We tracked the binding process and mechanism of occlusion by crystal soaking procedures.

### Structural rearrangements following Rb^+^ binding

#### Preoccluded (Rb)E2–BeF_x_ states

**Figure 6:**
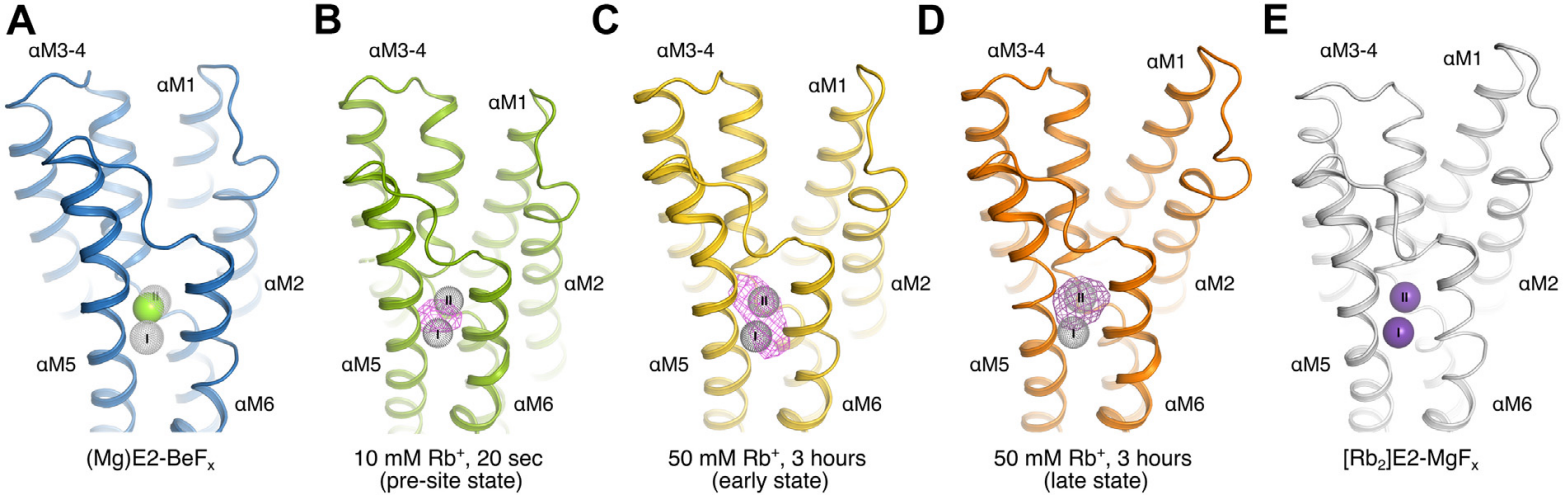
**Structural rearrangements following Rb**^**+**^**binding to E2–BeF**_**x**_**.***A*, open E2–BeF_x_ Mg^2+^-bound form (this study). Mg^2+^ is shown as a *green sphere*, and the positions of site I and II are indicated by *gray dotted spheres*. *B*, initial binding form (10 mM Rb^+^, 20 s). The 4-σ anomalous difference map is shown as a *purple mesh*, and cation-binding sites are shown as in *A*. Mg^2+^ site from open E2–BeF_x_ state is shown as *green dotted sphere*. *C*, early (Rb)E2–BeF_x_ form (soaked with 50 mM for 3 h, protomer 1). The 4-σ anomalous difference map is shown as a *purple mesh*, and cation-binding sites are shown as in *A*. *D*, late (Rb)E2–BeF_x_ form (soaked with 50 mM for 3 h, protomer 2). The anomalous difference map is contoured at 3σ (*red mesh*), and cation-binding sites are shown as in *A*. *E*, occluded [Rb_2_]E2–MgF_x_ form (Protein Data Bank [PDB] ID: 3KDP) (6). Rb^+^ ions are shown as *purple spheres*.

Structural rearrangements in the BeF_x_ complex because of Rb^+^ binding and occlusion were followed by soaking E2–BeF_x_ crystals. Two datasets were collected at 6.9 and 7.5 Å maximum resolution (consisting of 11,991 and 9616 unique reflections, respectively) at a wavelength of 0.814 Å with a strong anomalous signal for Rb^+^. They represent transition intermediates from the native E2–BeF_x_ with bound Mg^2+^ (Fig. 6*A*) toward the earlier reported occluded structure of [Rb_2_]E2–MgF_x_ (PDB ID: 3KDP (6)). Indeed, the datasets for Rb^+^-soaked E2–BeF_x_ (here referred to as quick soak and long soak corresponding to 10 mM, 20 s and 50 mM, 3 h, respectively) produced strong anomalous difference peaks (5.8–8.4 σ) at the cation-binding sites, reflecting exchange of Mg^2+^ with Rb^+^ (Fig. 6, *B*–*D*). As the asymmetric unit of the P2_1_2_1_2_1_ crystals consists of two protomers, we have altogether four representations of Rb^+^-soaked E2–BeF_x_ states (see crystal packing, Figs. S3 and S4).

Quantitative determination of Rb^+^ occupancy was challenging because of the low resolution of the datasets, but occupancy analyses through molecular replacement with single-wavelength anomalous diffraction refinement (MR-SAD, see Experimental procedures section) appeared consistent with two fully occupied binding sites for both protomers in the long Rb^+^ soak (occupancy > 0.75). For the two quick soak conformations, one protomer was fully occupied, whereas the other showed partial occupancy (with an occupancy less than 0.1 for a second site, assuming a full occupancy of a first site). Interestingly, the anomalous difference peak for the partially occupied quick soak protomer overlapped with the Mg^2+^ site between the two K^+^ sites I and II, suggesting that initial Rb^+^/K^+^ binding takes place at this exposed site prior to formation of the properly coordinated K^+^ (or Rb^+^) sites (Fig. 6*B*). That said, the low resolution obviously precludes firm conclusions on this point, but it should be noted that many thousand reflections contribute to the derived values.

Rigid-body model refinement for the cytosolic domains and individual TM segments produced large improvements in crystallographic *R*-factors for model representations and converged robustly at conformational changes that were also consistent with unbiased omit map controls.

For all four protomers of the soaked crystals, the cytoplasmic domains exhibited weak density in the electron density maps, likely indicating flexibility (Figs. S5 and S6). The E2–BeF_x_ MR model provided the relative position of the cytosolic headpiece based on some defined helices in each domain; thus, they are still modeled despite the weak density. The TM helices, however, were clearly visible in the electron density map to model the C_α_ main chain. The two protomers in the quick soak are similar to each other (rmsd = 1.16 Å, all C_α_; Fig. S5) and also show resemblance to protomer 1 in the long-soaked crystal (rmsd: ∼1.27 Å, all C_α_). However, the last protomer (long soak protomer 2) has a different conformation for the TM helices (rmsd = 1.96 Å between C_α_ of the two long soak protomers; Fig. S6). For simplicity, the protomers will therefore in the following be referred to as the initial (Rb)E2–BeF_x_ binding form (quick soak conformations), the early (Rb_2_)E2–BeF_x_ binding form (long soak protomer 1), and the late (Rb_2_)E2–BeF_x_ binding form (long soak protomer 2) when discussing structural changes.

#### Sequence of events

The refined atomic models for the E2–BeF_x_ form (this study) and [Rb_2_]E2–MgF_x_ form (6) represent the start and end points of the K^+^ binding, and the rigid body refined models of the soaked crystals, albeit determined at low resolution, provide valuable insights into the trajectories of the K^+^/Rb^+^-induced conformational changes that activate the dephosphorylation reaction.

**Figure 7:**
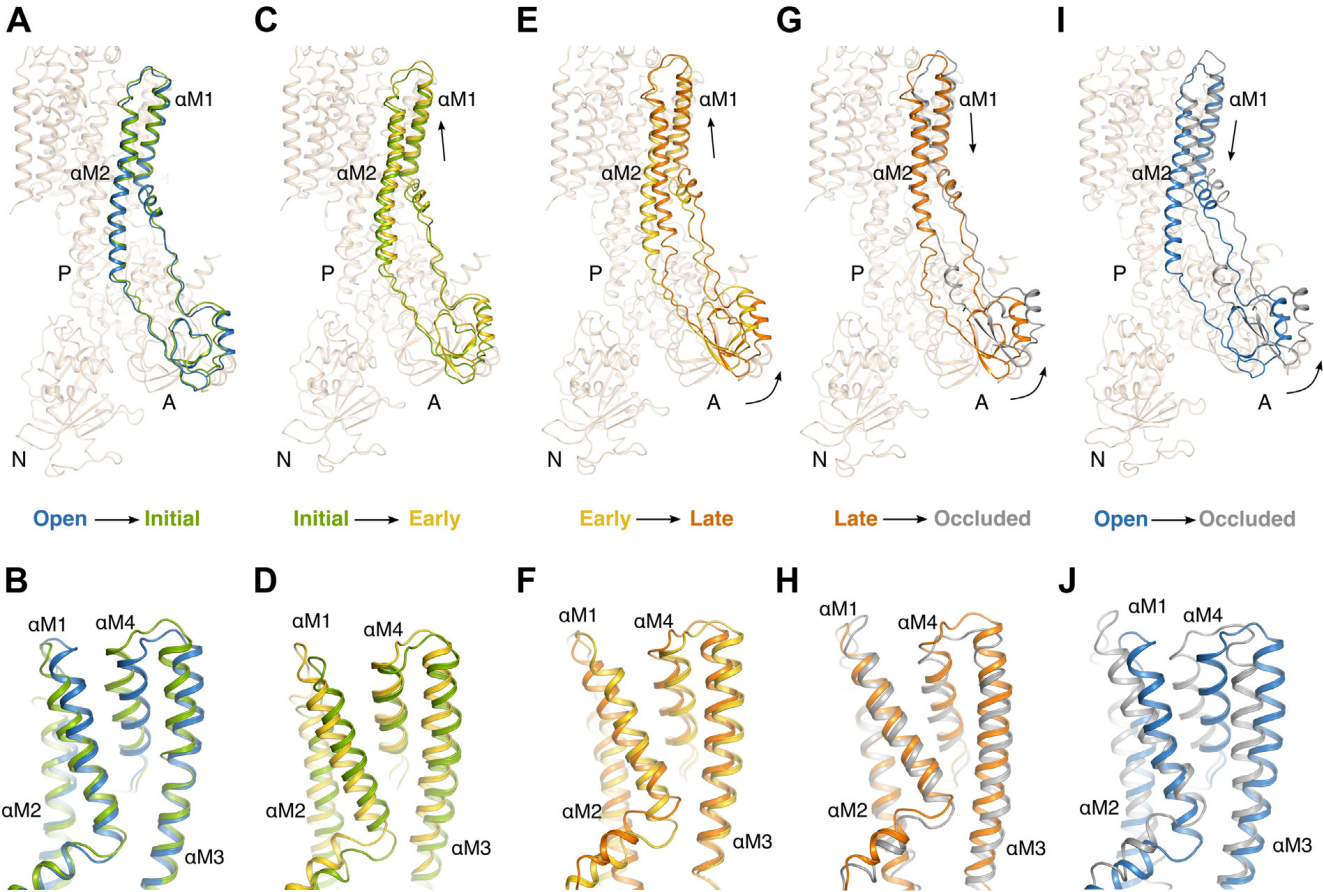
**Sequential structural changes leading to occlusion of Rb**^**+**^**in the E2 state.** Structural differences (*A* and *B*) between the open E2–BeF_x_ Mg^2+^ (*blue*) and initial binding (Rb)E2–BeF_x_ form (*green*) in an overall view (*A*) and for the transmembrane domain (*B*), respectively, (*C* and *D*) the initial (Rb)E2–BeF_x_ form (*green*) and the early (Rb)E2–BeF_x_ (*gold*), (*E* and *F*) early (Rb)E2–BeF_x_ (*gold*) and late (Rb)E2–BeF_x_ form (*orange*), (*G* and *H*) late (Rb)E2–BeF_x_ form (*orange*) and fully occluded [Rb_2_]E2–MgF_x_ (*gray*) (Protein Data Bank [PDB] ID: 3KDP (6)), and (*I* and *J*) open E2–BeF_x_ Mg^2+^ (*blue*) and fully occluded [Rb_2_]E2–MgF_x_ (*gray*). All structures were aligned on αM7–M10. For clarity, only the regions showing major conformational rearrangements between the states have been highlighted.

Superposition of the Rb^+^-soaked structures and the native E2–BeF_x_ form based on the TM αM7–M10 segment revealed sequential closing movements of the M1–M4 helices (Fig. 7). K^+^ binding associates the extracellular part of αM4 to site II, most likely through main chain carbonyls of Val322, Ala323, and Val325 engaging in coordination (6, 22). In the initial binding (Rb)E2–BeF_x_ form, this results in αM4 tilting ∼7° toward αM6 (Fig. 7, *A* and *B*). In the early (Rb_2_)E2–BeF_x_ form, αM4 is further tilted ∼5°, and the extracellular gate is closing. Through van der Waals contacts between αM1, αM2, and αM4, the αM1–M2 segment follows αM4 to an intermediate position *via* a ∼2 Å translation toward the extracellular side (Fig. 7, *C* and *D*).

In the next step, reaching the late (Rb_2_)E2–BeF_x_ form, the αM1–M2 helices are further translated toward to extracellular side (∼4.5 Å) and ∼6° lateral tilted toward the cytosolic side. The cytoplasmic part of the αM2 helix bends toward the A-αM3 linker region (Fig. 7, *E* and *F*). The change of path is likely realized by a partial unwinding of the αM2 helix (M2 switch (23)), which also gains flexibility; as is indicated by poor density for the cytoplasmic end of αM2 (Fig. S6). The unwinding enables the A domain to rotate ∼7 ° around the phosphorylation site of the P domain (toward the membrane). To complete the transition to the fully occluded [Rb_2_]E2–MgF_x_ complex (PDB ID: 3KDP, (6)), the A domain must finish its rotation (∼7°), bringing the TGES motif into dephosphorylation mode. This rotation causes a further ∼1.5 Å translation of the αM1–M2 segment toward the cytoplasmic side and a further unwinding of the αM2 cytoplasmic end between Glu144 and Ile150. Stabilizing the fully occluded state, the segment Ile150–Lys155 rewinds to form a hydrophobic cluster (24, 25) that ensures tight association between αM2-A and the A-αM3 linker segment with the P domain (Fig. 7, *G* and *H*).

Interestingly, similar sequential rearrangements are seen for the SERCA1a E2–BeF_x_ to E2–MgF_x_ transition when comparing thapsigargin-free and thapsigargin-bound (and proton-occluded) SERCA1a E2–BeF_x_ structures (PDB IDs: 3B9B and 2ZBE *versus* 2ZBF (15, 23)) (Figs. S7 and S8). This indicates that ion transporting P2-type ATPases (including H^+^,K^+^-ATPase (26, 27)) undergo similar conformational changes in dephosphorylation, yet differing from, for example, P1B-ATPases (28) and P4-ATPases (29, 30).

#### Changes in the α-C-terminal region and β-subunit

The α-C-terminal region plays an important role in the transport cycle (6, 31, 32). Mutations in the region are associated with neurological disorders, and the C terminus seems integral to the function of the Na^+^ site III and protonation of K^+^-bound states (31). However, when comparing the E2–BeF_x_ and [Rb_2_]E2–MgF_x_ structures in a local αM8 helix superimposition, only subtle changes are seen. Although they may affect the position and dynamics of the conserved C-terminal tyrosine residue (Fig. S9*A*) and thereby solvent access and protonation of the Na^+^ site III, functionally important features are difficult to qualify from the current study. Similarly, the β-subunit TM helix (β-TM) makes a small lateral shift toward αM7 (∼2°) at Rb^+^ occlusion.

On the other hand, the flexible N-terminal tail of the β-subunit (Phe15–Ser31 modeled) undergoes a different twist in the Rb^+^-occluded state, changing its interaction with the α-subunit. In the E2–BeF_x_ state, βArg27 likely forms a salt bridge with Glu1013 (αM10′), which gets disrupted in the Rb^+^-occluded state, where βArg27 is instead exposed to the cytosol. The N-terminal tail of the β-subunit appears more extended, as βAsn18 (the resolved β-N terminus of [Rb_2_]E2–MgF_x_) is shifted 6.5 Å compared with E2–BeF_x_, in response to changes in position of the cytoplasmic domains of the α-subunit in the E2P-E2·P_i_ transition (Fig. S9*B*).

### **Binding of ions and nucleotides induces dissociation of BeF**_**x**_**from the Na**^**+**^**,K**^**+**^**-ATPase**

**Figure 8:**
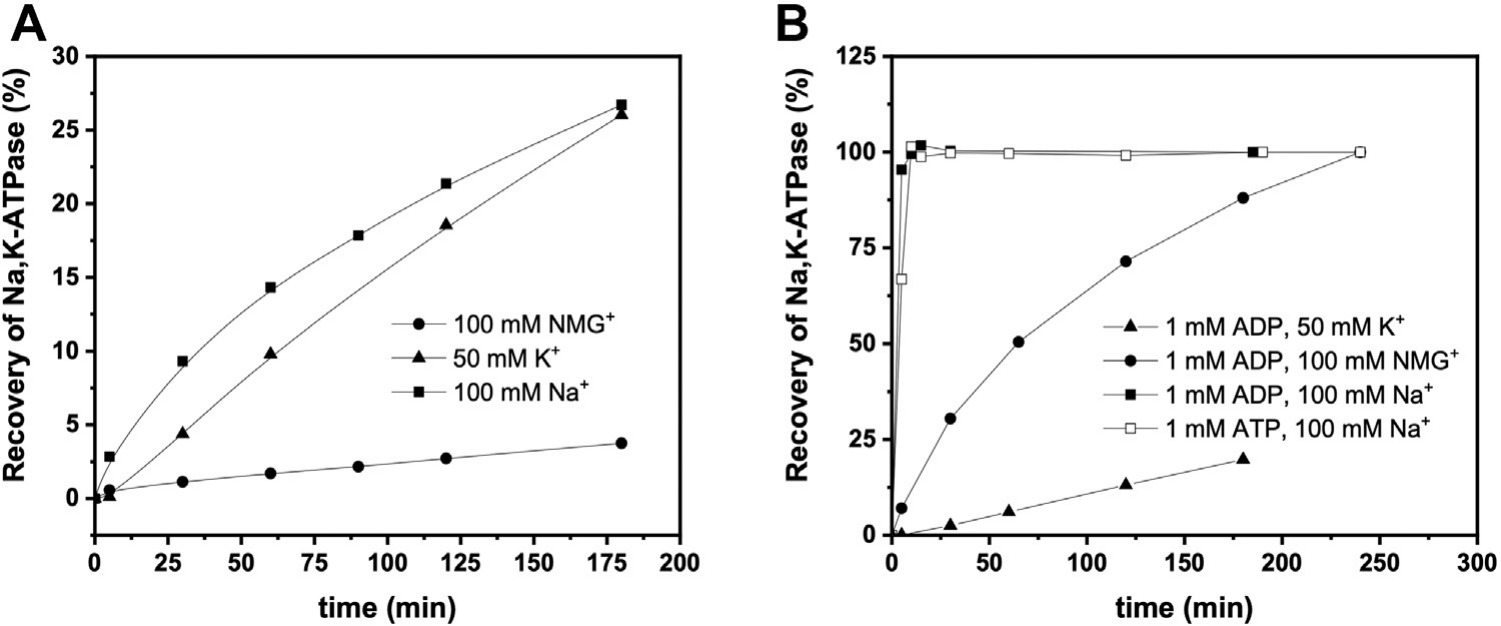
**Restoration of the Na**^**+**^**,K**^**+**^**-ATPase activity due to dissociation of BeF_x_**. (*A*) Time course of BeF_x_ dissociation from the Na^+^,K^+^-ATPase induced by cations. Note, that the Na^+^ concentration is twofold higher than that of K^+^. Total ion concentration is always 100 mM, adjusted with NMG^+^ when necessary (*B*). Time course of BeF_x_ dissociation induced by nucleotides and cations.

Monitoring the recovery of Na^+^,K^+^-ATPase activity, we investigated the effect of ions and nucleotides on the E2–BeF_x_ complex (Fig. 8). The time course of the reactivation of ATPase activity reflects the rate of BeF_x_ dissociation from the enzyme. Spontaneous dissociation in the media with 100 mM NMG^+^ (included to compensate for possible effects of ionic strength) is slow but increases in the presence of specific ions (Fig. 8*A*). K^+^ seems to be more efficient than Na^+^, as would be expected for extracellular ion binding. Note that Na^+^ concentration in the experiment is twofold higher than that of K^+^, whereas total ion concentrations are always 100 mM, adjusted with NMG^+^.

Also ADP (and ATP, but ADP formation during preincubation with ATP cannot be avoided) speeded up reactivation of the ATPase activity compared with NMG^+^ alone, and this effect is further amplified by Na^+^. On the other hand, K^+^ completely cancels the ADP effect, presumably by preventing its binding by allosterism.

Interestingly, the rate of dephosphorylation of the P_i_-induced E2P form does not increase upon addition of neither ADP nor K^+^ (21, 33), so our data point to an apparently significant difference between the two otherwise analogous conformations (P_i_-induced E2P and E2–BeF_x_). The explanation is, however, relatively straightforward: high stability of the E2–BeF_x_ form allows both ions and nucleotides to equilibrate with the protein and to influence and stimulate BeF_x_ dissociation. The acyl-phosphate bond on the other hand is reactive, and P_i_-induced E2P dephosphorylates with the rate constant of approximately 60 min^−1^ (21), that is, faster than equilibration with other ligands.

### **Docking of ADP into E2–BeF**_***x***_***structure***

**Figure 9:**
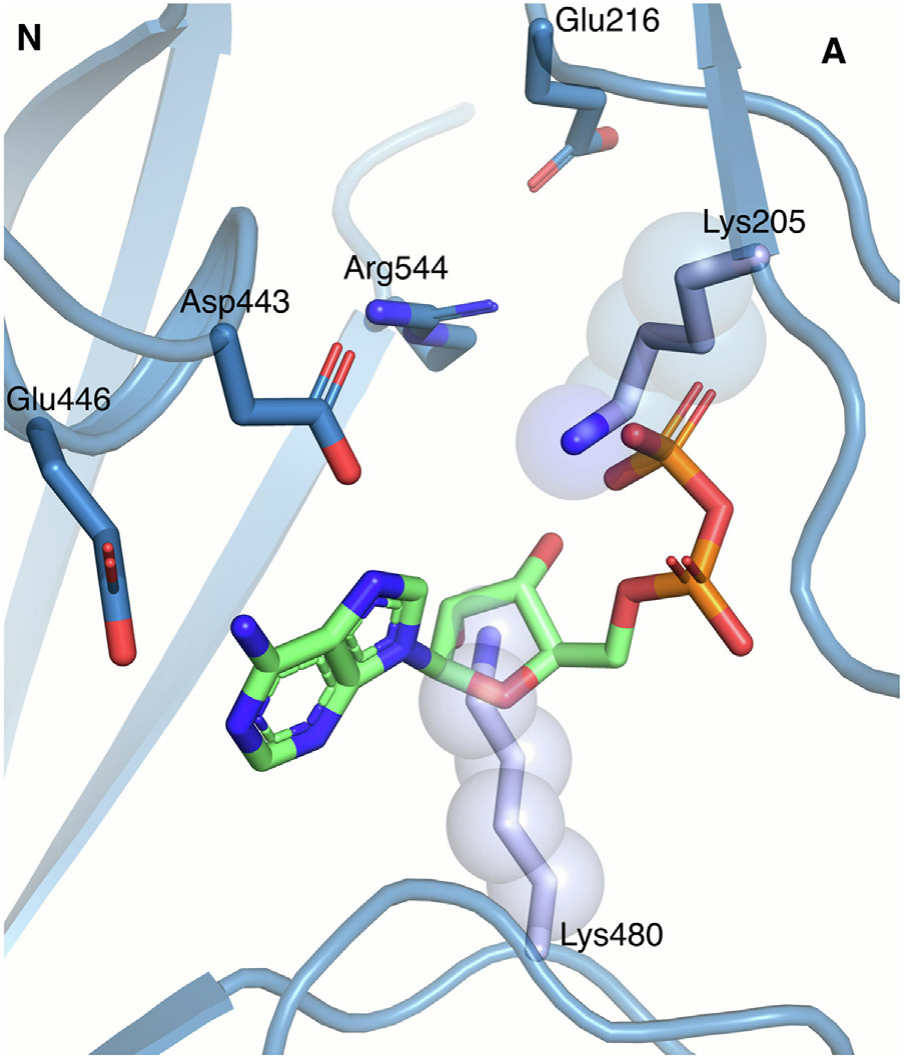
**ADP from the [Na**_**3**_**]E1–AlF**_**4**_^**−**^**-ADP structure (Protein Data Bank [PDB] ID:****3WGV** (34)**) modeled into the E2–BeF**_**x**_**complex.** N-domain alignment of the E2–BeF_x_ and the [Na_3_]E1–AlF_4_^−^-ADP structures showed a good fit for ADP at the nucleotide site with only few readjustment needed.

Visualization of ADP interaction with E2–BeF_x_ was approached by docking the ADP molecule from the [Na_3_]E1–AlF_x_–ADP structure (PDB ID: 3WGV (34)) into the E2–BeF_x_ state guided by a local structural alignment of the N domains. ADP fits well into the N domain of the E2–BeF_x_ complex (with Asp443 and Glu446 within coordination distance) and requires only minor rearrangement for the Phe475–Gln482 loop (Fig. 9*A*). The A domain comes into close contact with Lys205 near the β-phosphate of ADP (<1.9 Å), which presumably will cause a slight repositioning of the A domain. In the E2–MgF_x_ state, the interface between the N and A domains is only stabilized by an ionic bond between Arg544 (N domain) and Glu216 (A domain). Thus, it is likely that Arg544 either interacts with the β-phosphate of ADP upon its binding to E2–BeF_x_ (and allows further interaction with Na^+^) or forms a salt bridge with Glu216 as consequence of interactions with K^+^. Both destabilizing effects are likely to provide greater mobility of the A domain and accelerate BeF_x_ dissociation.

## Discussion

Comparison of the functional properties of the P_i_-induced E2P and E2–BeF_x_ revealed close similarities of these forms: kinetics of interactions with the cardiotonic steroids and K^+^ are virtually the same, and the structures of E2–BeF_x_ and E2P stabilized by cardiotonic steroids show clear structural resemblance.

The cation-binding sites are open to the extracellular side and accept Na^+^, K^+^, as well as Mg^2+^ at low affinity. The ability of E2–BeF_x_ to occlude Na^+^ is lost, even in the presence of oligomycin, and Na^+^ affinity is therefore low. Na^+^ binding, however, destabilizes E2–BeF_x_, in a way analogous to the dephosphorylation under the Na^+^/Na^+^ exchange reaction associated with ATP hydrolysis (35).

Binding of K^+^ (or the congener Rb^+^) triggers the conformational rearrangements necessary for ion occlusion and destabilization of the Asp369–BeF_x_ bond. Rb^+^ soaking of the E2–BeF_x_ crystals revealed a sequence of Rb^+^ preocclusion steps. Although only qualitative, the occupancy analysis revealed that the initial (Rb)E2–BeF_x_ binding form has only partial occupation at site I. Furthermore, the anomalous difference suggests an overlap of the initial site with the Mg^2+^ site (Fig. 6*B*). This indicates that going from the E2–BeF_x_ open state, K^+^ will bind in a sequential manner by first binding to an initial site that likely overlaps with the low-affinity Mg^2+^ site coordinated by Asn776, Glu779 (αM5), and Asp804 (αM6) (10). Subsequently, the initial site changes configuration to the K^+^ site I, at the same time making space for ion binding at site II. That involves main-chain carbonyls of αM4 and ultimately leads to closure of the extracellular gate. This mechanism, described from the outward-open E2–BeF_x_ form and onward, is consistent with exchange kinetics followed in the opposite direction. Thus, in the occluded [K2]E2–MgF_x_ form, the exchange with extracellular ions occurs first at site II (36). The oscillations of αM3–M4 helices allowing only one ion to bind at the time stand for the “flickering gate,” first proposed by Forbush (37) on the basis of deocclusion experiments and later supported by high-speed voltage-clamp measurements (16).

In summary, the conformational transitions observed in soaking experiments are the prelude to the dephosphorylation process in the Na,K-ATPase reaction. The sequential binding of two K^+^ through the αM1–M2-triggered rotation of the A domain rearranges the TGES motif, allowing water access and desphosphorylation (Fig. S10).

The aforementioned interactions with K^+^ suggest that the E2–BeF_x_ complex is a mimic of the ground state E2P. However, E2–BeF_x_ is also destabilized by ADP, that is, it has a property of an ADP-sensitive state (Fig. 8*B*). The ability of ADP to induce a direct hydrolysis of the acyl-phosphate bond was described earlier by Hobbs *et al.* (4) for the phosphoenzyme E∗P. ADP-induced dephosphorylation of E∗P occurred directly, without transition to E1P. In the case of E2–BeF_x_, however, a transition toward E1–BeF_x_ might be induced by the simultaneous presence of ADP and Na^+^, since the synergy between these two ligands is very clear (Fig. 8*B*). K^+^, on the other hand, cancels the ADP effect (Fig. 8*B*). Because of occlusion, it prohibits ADP binding and leads to slow BeF_x_ dissociation from the [K_2_]E2–BeF_x_ complex.

In conclusion, the E2–BeF_x_ complex of Na^+^,K^+^-ATPase represents a phosphorylated intermediate that can reach both E2P and E∗P. The structural similarity to the backdoor phosphorylated (P_i_-induced) E2P form is obvious from its comparison with the CTS-stabilized complexes of the Na,K-ATPase. Functionally, P_i_-induced E2P and E2–BeF_x_ react nearly identically with CTS (here demonstrated by anthroylouabain interactions) and with K^+^ (probed by RH421 fluorescence), and both forms are capable of Rb^+^ or K^+^ occlusion. Soaking of the E2–BeF_x_ crystals with Rb^+^ allowed visualization of different steps in the occlusion process. At the same time, firm coordination of BeF_x_ within the phosphorylation site grants time for nucleotide binding and exploration of a dynamic E∗P intermediate, unlike for E2P. These studies should be very informative for future investigations of P-type ATPase dynamics and studies of, for example, mutant forms.

## Experimental procedures

### Protein preparation

Pig kidney Na^+^,K^+^-ATPase was purified as previously described (38). The specific ATPase activity of the Na^+^,K^+^-ATPase-purified membrane preparations was about 1800 μmol P_i_ per hour per mg of membrane protein at 37 °C.

### Crystallization and data collection

The BeF_x_-inhibited complex of Na^+^,K^+^-ATPase was formed by preincubation of the membraneous enzyme in 20 mM histidine (pH 7.0), 10 mM NaF, 0.5 mM MgCl_2_, and 20 μM BeSO_4_. The stabilized E2–BeF_x_ complex was subsequently solubilized in the same buffer with the nonionic detergent octaethyleneglycol mono-*n*-dodecylether [C_12_E_8_] at a ratio of 0.9 mg C_12_E_8_ per mg protein, and insoluble material was removed by ultracentrifugation. The final concentration of solubilized protein was 9 to 10 mg/ml.

Crystals were grown by vapor diffusion at 15 °C in 2 μl hanging drops for 2 to 3 weeks. The protein sample was mixed in a 1:1 ratio with reservoir solution containing 16.5% (w/v) polyethylene glycol 2000 monomethyl ether, 10% (v/v) glycerol, 175 mM MgCl_2_, 150 mM NaCl, 20 mM Hepes/Mes (pH 6.2) and 2 mM DTT. The crystals were dehydrated overnight at 4 °C against a 30% polyethylene glycol 2000 monomethyl ether reservoir solution before flash cooling. The final datasets were collected at 100 K on the DESY-EMBL beamline P14 and the SLS-X06DA (PXIII) beamline. For Rb^+^ soaks, 1.1 mM sucrose monodecanoate was added to the solubilized protein before mixing with reservoir solution. About 10 and 50 mM RbCl, respectively, was added to the crystallization drop and equilibrated for 20 s and 3 h, respectively. The final datasets of Rb^+^-soaked crystals were collected on the DESY-EMBL beamline P13 and the DLS-I24 beamline using a wavelength of 0.814 Å.

### Data processing

Datasets were processed and scaled using XDS software (Wolfgang Kabsch, MPI for Medical Research) (39). The crystals showed P2_1_2_1_2_1_ space group symmetry with unit cell dimensions, *a* = 116.3 Å, *b* = 117.8 Å, and *c* = 490.9 Å and two αβγ heterotrimers per asymmetric unit. For the native E2–BeF_x_ structure, datasets derived from two crystals were merged to yield the final dataset. The data were anisotropically scaled using the Diffraction Anisotropy Server (http://services.mbi.ucla.edu/anisoscale) (40), setting the resolution limits to 5.4, 4.4, and 4.0 Å along a∗, b∗, and c∗, respectively. MR was performed using PHASER (Phenix supported program) (41). As a search model, we used the crystal structure of Na^+^,K^+^-ATPase E2P OBN complex (PDB ID: 4HYT (10)) and later also the structure of a different crystal form of the E2–BeF_x_ complex (PDB ID: 7D91) (9). Rigid body refinement followed by simulated annealing refinement protocol was performed in PHENIX (Phenix Industrial Consortium) (42). Manual refinement was carried out in Coot (MRC-LMB) (43), and the further model refinement was continued in PHENIX using noncrystallographic symmetry (NCS), translation–libration–screw parameterization, and grouped atomic displacement parameter refinement. Because of low resolution of the data, tight geometry restraints were imposed on the model to stabilize the refinement. Rigid body groups were defined by the A, N, and P domains along with the αM1–2, αM3–4, αM5–10/βM/γM, and β-ectodomain. NCS and translation–libration–screw groups were defined by the A, N, and P domains, the TM domain αM1–10/βM/γM, and the β-ectodomain. The quality of the final model was assessed using the MOLPROBITY server (Duke Biochemistry, Duke University School of Medicine) (44). For Rb^+^-soaked datasets, the diffraction data were first scaled using ScaleIT (CCP4 supported program) (45) to the Na^+^,K^+^-ATPase E2P OBN complex (PDB ID: 4HYT) (10), and the initial phases were obtained by MR using PHASER and the E2–BeF_x_ structure. Rigid body refinement, NCS, and atomic displacement parameter refinement (same groups as for the E2–BeF_x_ structure) were performed for each dataset. Occupancy calculations were performed using MR with single-wavelength anomalous diffraction (46). All protein structure figures were prepared using PyMOL (The PyMOL Molecular Graphics System, version 2.3.0 [Schrödinger LLC, 2012]).

### Na^+^ occlusion

The membrane-bound enzyme (0.5 mg/ml) was incubated with 1 mM ^22^NaF in the presence of 10 mM Tris–HCl (pH 7.0), 0.5 mM MgCl_2_, 1 mM ^3^H-glucose in the presence or the absence of 0.02 mM BeSO_4_, with or without 0.02 mg/ml oligomycin for 5 h at 0 °C. Then, 0.4 ml of the mixture was loaded on a Millipore HAWP 0.45 μm filter and immediately washed with 1 ml of Tris–HCl buffer containing 20 mM NaCl to decrease background. The filters were counted in 4 ml Packard Filtercount scintillation fluid. ^3^H-counts gave an estimate for the nonspecific absorption of the isotopes of the filter and for the amount trapped in the wetting volume.

### Dissociation of BeF_x_ from the Na^+^,K^+^-ATPase induced by different ligands

BeF_x_ complex of Na^+^,K^+^-ATPase was formed by preincubation of the enzyme in 20 mM histidine (pH 7.0), 10 mM NaF, 0.5 mM MgCl_2_, and 20 μM BeSO_4_ on ice overnight. The suspension was subjected to centrifugation 30 min × 50,000*g* at 4 °C, and the pellet was resuspended in the media containing 20 mM histidine (pH 7.0) and varying ligands in concentrations as noted in Figure 8. After different periods of incubation at 37 °C, the aliquots from each sample were used for measurement of the Na^+^,K^+^-ATPase activity under optimal conditions (47).

### Fluorescence spectroscopy

#### RH421 fluorescence experiments

Equilibrium and transient RH421 fluorescence in response to varying concentrations of KCl was measured at room temperature in 20 mM histidine (pH 7.0), 10 mM NaF, 0.5 mM MgCl_2_, 0.05 mg/ml enzyme, and 0.2 μg/ml RH421 (VWR). Formation of BeF_x_ complex was ensured by addition of 20 μM BeSO_4_. Measurements under equilibrium conditions were performed on a SPEX Fluorolog fluorometer in a cuvette with 1 cm light path with continuous stirring. Excitation wavelength was 580 nm (slit 2 nm) and emission 630 nm (slit 14 nm). Observed rate constants were obtained in the experiments using rapid-mixing stopped-flow spectrofluorometer (Applied Photophysics) at excitation wavelength of 580 nm (slit 1 nm) with 630 nm cutoff filter on the emission side.

#### Anthroylouabain fluorescence experiments

Equilibrium and transient experiments with anthroylouabain were performed at room temperature in either 20 mM histidine (pH 7.0), 4 mM H_3_PO_4_ (adjusted with *N*-methyl-d-glutamine), 4 mM MgCl_2_, 0.05 mg/ml enzyme (P_i_-induced E2P complex); or 20 mM histidine (pH 7.0), 10 mM NaF, 0.5 mM MgCl_2_, 20 μM BeSO_4_, and 0.05 mg/ml enzyme (BeF_x_ complex). When necessary, K^+^ was added as KCl in the experiments with P_i_-induced E2P complex form, whereas in the case of E2–BeF_x_ complex, 10 mM NaF was replaced by equimolar KF. A SPEX Fluorolog fluorometer was used to monitor slow reactions, excitation at 370 nm (slit 5 nm), emission at 485 nm (slit 5 nm). Association rate constants were obtained in the experiments using rapid-mixing stopped-flow spectrofluorometer at excitation wavelength of 370 nm (slit 1 nm) with 485 nm cutoff filter on the emission side.

Dissociation of anthroylouabain complexes was performed in a chase experiment as follows: the enzyme complexes were preincubated with 0.2 μM anthroylouabain overnight. Then 3.3 mM ouabain was added to the sample (anthroylouabain was diluted to 0.13 μM simultaneously), and decrease in fluorescence was monitored in time.

#### Two-electrode voltage clamping

The experiments were performed as described (48). In brief, mRNA was made with the mMESSAGE mMACHINE T7 Ultra Kit (Life Technologies) from linearized DNA encoding the human α2 with mutations to decrease OBN sensitivity (Q116R and N127D) and β1. *X. laevis* oocytes (EcoCyte Bioscience) were injected with 9.5/2.5 ng α2/β1 subunit mRNA in 50.6 nl. Oocytes were incubated 2 to 8 days at 12 to 18 °C in ND96 buffer supplemented with 25 μg/ml gentamicin and 2.5 mM sodium pyruvate. Oocytes were clamped with an OC-725C amplifier (Warner Instruments), and the signal was digitized by a 1440A digitizer (Molecular Devices). Data were recorded with PClamp 10.4 (Molecular Devices) and analyzed with ClampFit (Molecular Devices) and GraphPad Prism (GraphPad Software, Inc). For sodium/sodium exchange recordings, the extracellular buffer contained 115 mM NaOH, 110 mM sulfamic acid, 10 mM Hepes, 5 mM BaCl_2_, 1 mM MgCl_2_, 0.5 mM CaCl_2_, 10 μM OBN, pH 7.4 (adjusted with sulfamic acid). For other recordings, NaOH was replaced by NMDG or KOH as indicated. Voltage jumps were performed from a holding potential of −50 mV with 200 ms jumps to potentials between 60 mV and −160 mV in 20 mV steps. Charge (Q) and rate currents were determined by subtracting a recording with 10 mM OBN added from the immediately preceding recording. On- and off-currents were fitted with single exponentials to determine rate currents and charge, respectively. Charges were fitted to a Boltzmann distribution. For evaluation of steady-state currents, the voltage protocol was performed in buffer, buffer with 10 mM OBN, and buffer. The last recording ensures stability throughout the recordings.

## Data availability

Coordinates and diffraction data for the E2–BeF_x_ structure and for the low-resolution models of the initial, early, and late stages of Rb^+^ binding have been deposited in the PDB under accession codes 7QTV, 7YZR, and 7Z04.

## Supporting information

This article contains supporting information (6, 15, 23, 50, 51).

## Conflict of interest

The authors declare that they have no conflicts of interest with the contents of this article.
