# Supplementary material for: The Na+,K+-ATPase in complex with beryllium fluoride mimics an ATPase phosphorylated state
Source: J Biol Chem. 2022 Aug 2;298(9):102317. doi: 10.1016/j.jbc.2022.102317 (PMC9485054; doi:10.1016/j.jbc.2022.102317)
Supplement: Supplemental Figures S1–S10 [file mmc1.docx]

Supporting information for:

**The Na^+^,K^+^-ATPase in complex with beryllium fluoride mimics an ATPase phosphorylated state**

Marlene U. Fruergaard^1^, Ingrid Dach^1,§^, Jacob L. Andersen^1,2,#^, Mette Ozol^1&^, Azadeh Shahsavar^1$^, Esben M. Quistgaard^1€^, Hanne Poulsen^1%^, Natalya U. Fedosova^2*^, Poul Nissen^1*^

^1^DANDRITE - Nordic EMBL Partnership for Molecular Medicine, Aarhus University, Dept. Molecular Biology and Genetics, DK – 8000 Aarhus C, Denmark

^2^Department of Biomedicine, Aarhus University, DK – 8000 Aarhus C, Denmark

^*^Correspondence: [nf@biomed.au.dk](mailto:nf@biomed.au.dk) or [pn@mbg.au.dk](mailto:pn@mbg.au.dk)

^§^Current address: Otoč Korunu, Czech Republic; ^#^Current address: Meelunie GPI, Hedensted, Denmark; ^&^Current address: Eurofins Laboratories, Galten, Denmark; ^$^Current address: Department of Pharmacology and Drug Design, University of Copenhagen, Denmark; ^€^Current address: Danish Medicines Agency, Copenhagen, Denmark; ^%^Current address: Department of Pathology, Aarhus University Hospital, Denmark


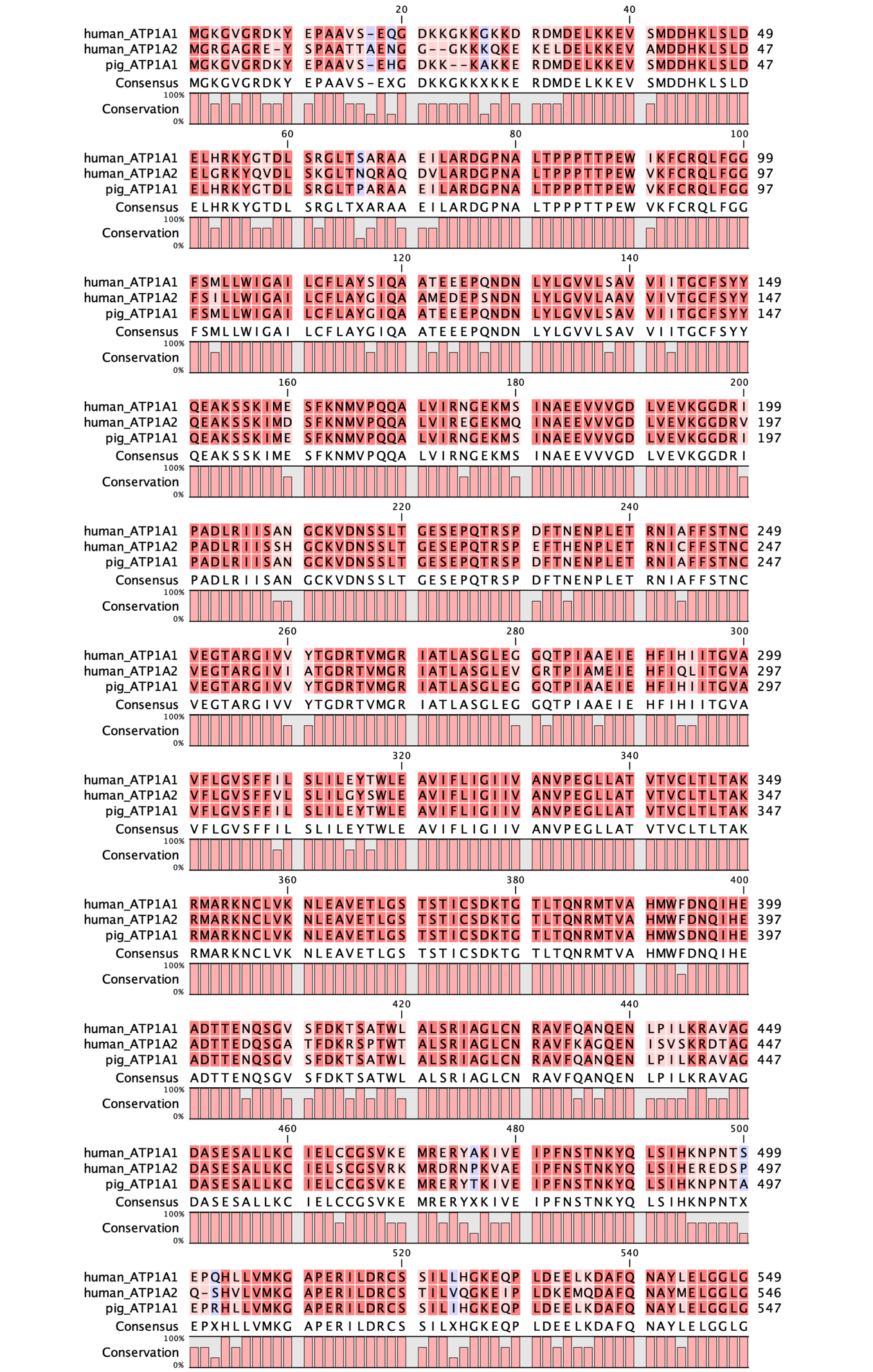

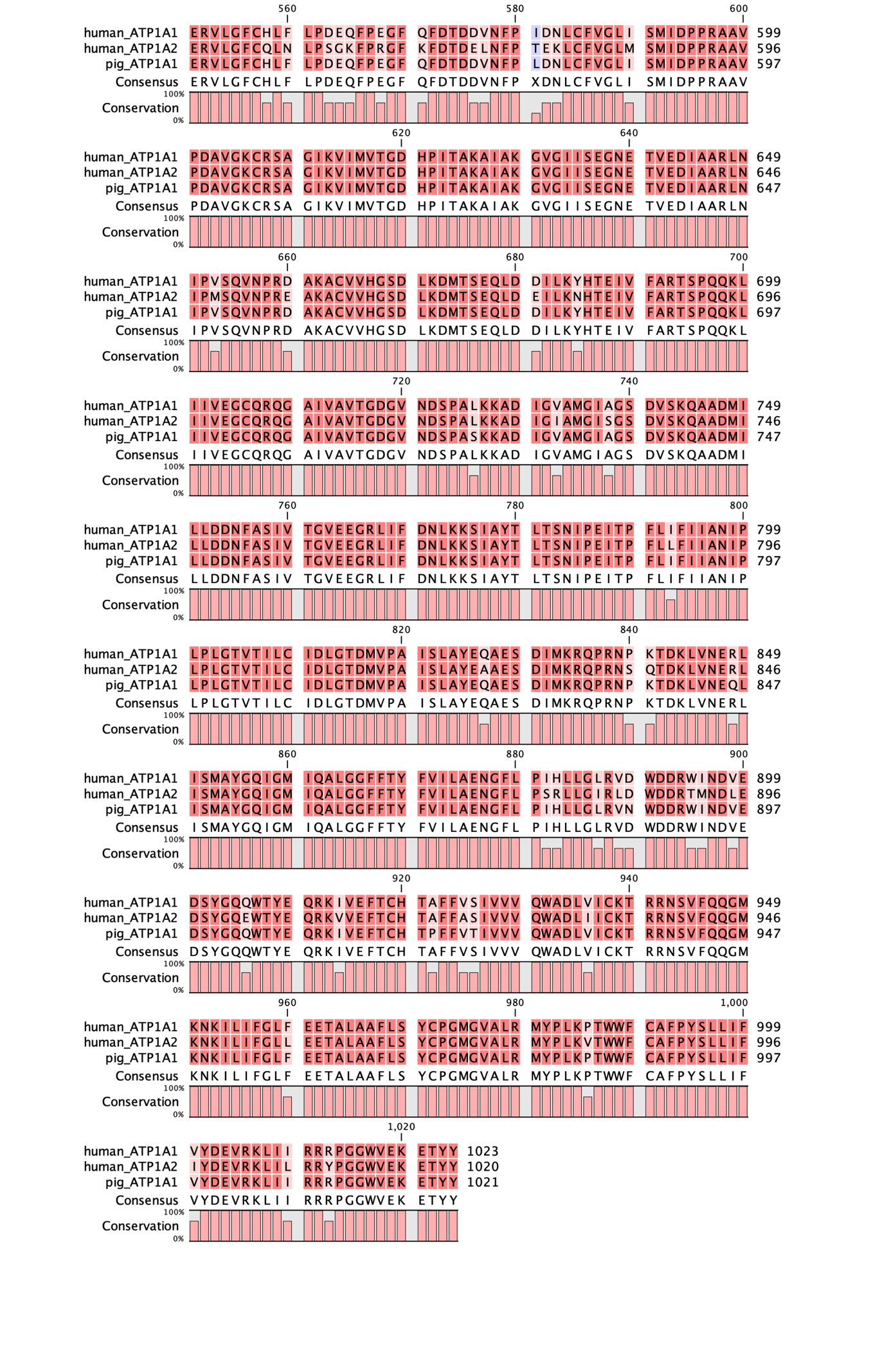


# Fig. S1. Sequence alignment of human ATP1A1 (α1), human ATP1A2 (α2), and pig ATP1A1 (α1), aligned by Clustal Omega(1). The shading indicates conservation (blue 0% - red 100 %). Uniprot identifiers: human ATP1A1 – P05023, human ATP1A2 – P50993, pig ATP1A1 – P05024.

**A**

**B**

**C**

**D**

**Fig. S2.** Magnesium effect on sodium and potassium binding. A) Voltage jumps under sodium/sodium exchange conditions in oocytes expressing human α2β1 (n=7). The ouabain-sensitive currents are determined at three magnesium ion concentrations for each oocyte. Q/V_m_ curves and B) rate constants were determined for each concentration of magnesium. C) The efficacy of potassium was tested at -50 mM and 100 mM potassium as the pumping current (I) in the presence and absence of 5 mM magnesium (n=4). Line at mean with SD. D) Potassium affinity was determined with 0 mM, 1 mM and 5 mM magnesium.

**
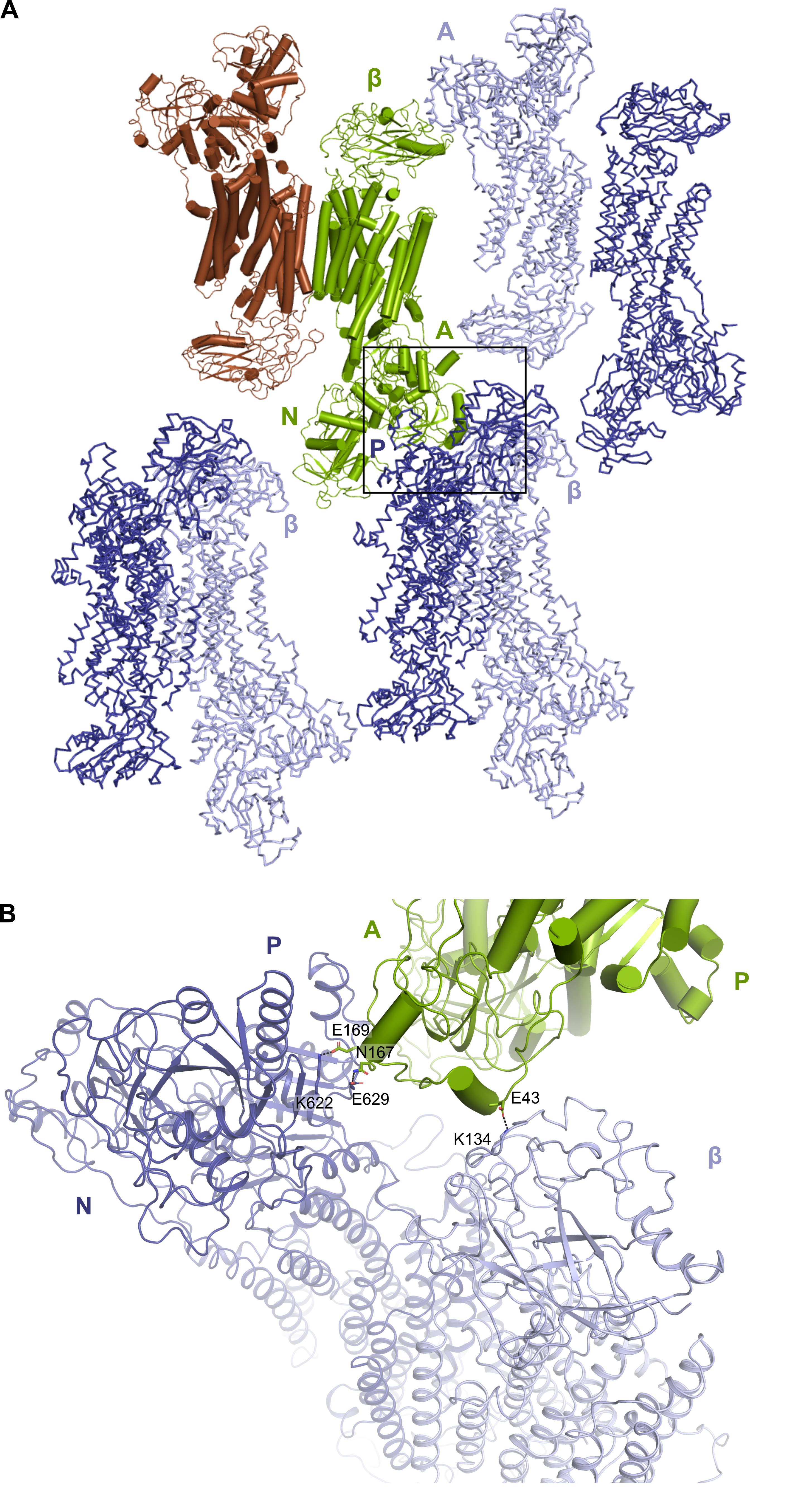
**

**Fig. S3.** P2_1_2_1_2_1_ crystal packing. (A) Cartoon representations of 10 mM Rb^+^ quick soak protomers (initial state) depicted in brown and green, respectively. Symmetry related protomers are shown as ribbon in light blue and dark blue, respectively. The A domain makes crystal contacts with a P domain and β ectodomain to nearby protomers. (B) Close-up view of areas boxed in (A) showing potential hydrogen bond interactions to nearby protomers.


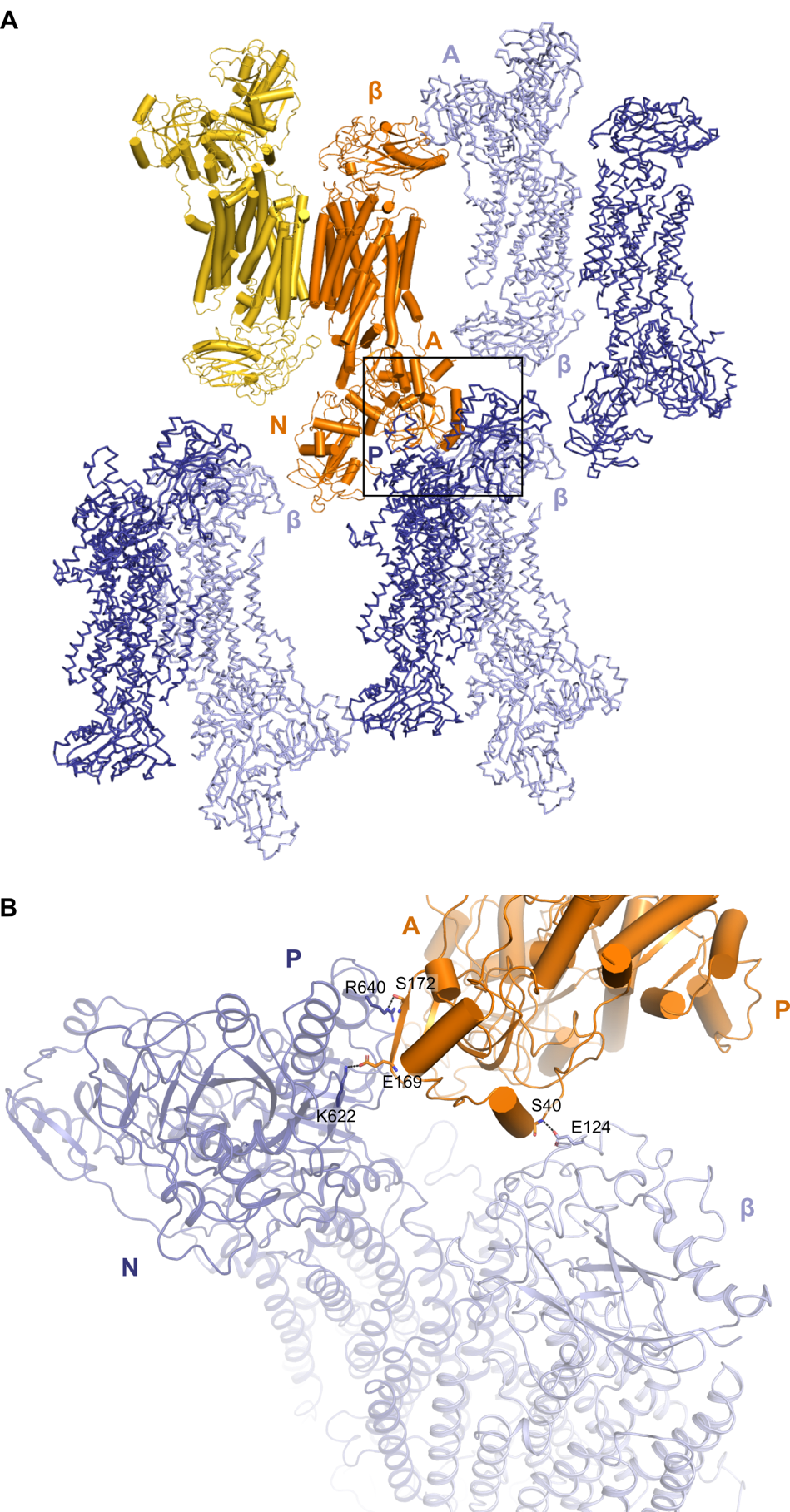


**Fig. S4.** P2_1_2_1_2_1_ crystal packing. (A) Cartoon representations of 50 mM long soak protomers (early and late states) depicted in yellow and orange, respectively. Symmetry related protomers are shown as ribbon in light blue and dark blue, respectively. The A domain makes crystal contacts with a P domain and β ectodomain to nearby protomers. (B) Close-up view of areas boxed in (A) showing potential hydrogen bond interactions to nearby protomers.





**Fig. S5.** (A,B) Electron density maps for two protomers of the (Rb)E2-BeF_x_ initial site form (10 mM Rb^+^, 20 sec). 2F_o_-F_c_ is depicted in gray mesh (contoured at 1.3 σ). (C) αM7-M10 transmembrane alignment of the two protomers showing high resemblance (root mean square deviations (rmsd) = 1.16 Å, all C_α_).





**Fig. S6.** Electron density maps for (A) the early (Rb)E2-BeF_x_ form (protomer 1) and (B) the late (Rb)E2-BeF_x_ form (protomer 2) from the 50 mM Rb^+^-soaked crystal (50 mM Rb^+^, 3 hour). 2F_o_-F_c_ is depicted in gray mesh (contoured at 1.3 σ). (C) αM7-M10 transmembrane alignment of the two protomers (rmsd = 1.96 Å, C_α_).





**Fig. S7.** Sequential structural changes from (A,B) the E2-BeF_x_ open state (blue) to (Rb_2_)E2-BeF_x_ form early (gold), (C,D) early form to (Rb_2_)E2-BeF_x_ late form (orange), and (E,F) late form to fully occluded [Rb_2_]E2-MgF_x_ state (gray) (PDB code 3KDP(2)). For clarity, domains P, N, αM3-M10, β- and γ- subunits for all structures (except E2-BeF_x_ open state) have been removed in the upper panel (A,C,E). Only the regions showing major conformational re-arrangements between the states have been highlighted.

**

**

**Fig. S8.** Sequential structural changes from (A,B) the SERCA E2-BeF_x_ open state (PDB ID 3B9B (3); cyan) to E2-BeF_x_ thapsigargin free-form (PDB ID 2ZBE (4); yellow), (C,D) from E2-BeF_x_ thapsigargin free-form to E2-BeF_x_ thapsigargin free-bound (PDB ID 2ZBF (4); red), and (E,F) from thapsigargin free-bound to E2-MgF_x_ (PDB ID 3FGO (5); green). For clarity, domains P, N and αM3-M10 for all structures (except E2-BeF_x_ open state) have been removed. Only the regions showing major conformational re-arrangements between the states have been highlighted.

**

**

**Fig. S9.** Local changes in the α-C-terminal region and β-subunit in the E2-BeF_x_ (blue) to [Rb_2_]E2-MgF_x_ (PDB 3KDP (2), grey) transition. (A) Flexibility at the C-terminal Tyr1016 associated with H^+^ leak currents. Structures are here aligned based on a local αM8 superimposition. Density map contoured at 1.5 σ. (B) Movement of the β-TM helix and its cytosolic N-terminal segment. The ionic interaction of Arg27 (β) and Glu1013 (αM10´) is disrupted upon Rb^+^ occlusion as the β N-terminal is changing its position due to movements of the cytoplasmic domains of the α-subunit in formation of the dephosphorylation complex. The structures were aligned as in A and density map is contoured at 1.5 σ.

**

**

**Fig. S10.** Consequences of K^+^ binding within the extracellular sites of the Na^+^,K^+^-ATPase (cartoon). The sequence of events starts with binding of one ion at the initial site (overlapping with the Mg^2+^ site), dragging αM4 towards M6. As the second ion binds, the closure of the gate, pushes the M1-M2 segment ~2 Å upwards towards the extracellular side through van der Waals contacts, which causes the A domain to rotate ~7º around the phosphorylation site (towards the membrane away from the N domain). Following a further small rotation of the A domain and αM1-M2 relocation, the dephosphorylation reaction becomes catalyzed by the TGES motif, which leads to the full occlusion of K^+^ ions ([K_2_]E2P_i_ state(2)).

**References**

1. Sievers, F., Wilm, A., Dineen, D., Gibson, T. J., Karplus, K., Li, W., Lopez, R., McWilliam, H., Remmert, M., Soding, J., Thompson, J. D., and Higgins, D. G. (2011) Fast, scalable generation of high-quality protein multiple sequence alignments using Clustal Omega. *Mol Syst Biol* **7**, 539

2. Morth, J. P., Pedersen, B. P., Toustrup-Jensen, M. S., Sorensen, T. L., Petersen, J., Andersen, J. P., Vilsen, B., and Nissen, P. (2007) Crystal structure of the sodium-potassium pump. *Nature* **450**, 1043-1049

3. Olesen, C., Picard, M., Winther, A. M., Gyrup, C., Morth, J. P., Oxvig, C., Moller, J. V., and Nissen, P. (2007) The structural basis of calcium transport by the calcium pump. *Nature* **450**, 1036-1042

4. Toyoshima, C., Norimatsu, Y., Iwasawa, S., Tsuda, T., and Ogawa, H. (2007) How processing of aspartylphosphate is coupled to lumenal gating of the ion pathway in the calcium pump. *Proc Natl Acad Sci U S A* **104**, 19831-19836

5. Laursen, M., Bublitz, M., Moncoq, K., Olesen, C., Moller, J. V., Young, H. S., Nissen, P., and Morth, J. P. (2009) Cyclopiazonic acid is complexed to a divalent metal ion when bound to the sarcoplasmic reticulum Ca2+-ATPase. *J Biol Chem* **284**, 13513-13518
